# Association of dynamic changes in metabolic syndrome components with clinical outcomes in diffuse large B-cell lymphoma

**DOI:** 10.3389/fonc.2025.1524498

**Published:** 2025-06-16

**Authors:** Dewan Zhao, He Xu, Fengrao Tang, Tongcheng Cui, Xiuli Sun, Guirong Song

**Affiliations:** ^1^ Department of Hematology, The First Affiliated Hospital of Dalian Medical University, Dalian, Liaoning, China; ^2^ Department of Health Statistics, School of Public Health, Dalian Medical University, Dalian, Liaoning, China

**Keywords:** body mass index, HDL cholesterol, LDL cholesterol, diffuse large B-cell lymphoma, metabolic syndrome

## Abstract

**Introduction:**

Diffuse Large B-Cell Lymphoma (DLBCL) is the most common subtype of Non-Hodgkin Lymphoma (NHL), with 20-40% of patients experiencing poor outcomes despite advancements in treatment. While Metabolic Syndrome (MetS) has been linked to NHL prognosis, its impact on DLBCL outcomes remains unclear.

**Methods:**

This study examined the effects of dynamic changes in MetS components on DLBCL treatment outcomes and prognosis. We retrospectively analyzed 125 newly diagnosed DLBCL patients treated with 6-8 cycles of CHOP (cyclophosphamide, doxorubicin, vincristine, and prednisone) or CHOP-like regimens, with or without rituximab, from May 2010 to May 2022. Group-based trajectory models were used to identify MetS component trajectories. Multivariate logistic regression and Cox proportional hazards regression were employed to determine factors affecting complete remission (CR), progression-free survival (PFS), and overall survival (OS).

**Results:**

The 2-year PFS and OS rates were 70.0% and 82.0%, respectively. High baseline high-density lipoprotein cholesterol (HDL-C) was associated with reduced progression risk (HR = 0.27, 95% CI: 0.10-0.78), while high baseline low-density lipoprotein cholesterol (LDL-C) was linked to decreased CR rate (OR = 0.65, 95% CI: 0.44-0.97) and increased progression risk (HR = 1.78, 95% CI: 1.14-2.79). Additionally, high LDL-C trajectory was associated with reduced CR rates, whereas moderate BMI trajectory was associated with improved CR, PFS, and OS.

**Discussion:**

Therefore, controlling LDL-C levels and maintaining a moderate BMI are crucial for improving DLBCL clinical outcomes.

## 1 Introduction

Diffuse Large B-Cell Lymphoma (DLBCL) is the most common subtype of Non-Hodgkin Lymphoma (NHL), accounting for 30%-40% of adult NHL cases worldwide (1) and 30-50% in China (2). Although the advent of rituximab-based immunochemotherapy, such as R-CHOP (rituximab combined with cyclophosphamide, doxorubicin, vincristine, and prednisone), has substantially improved survival outcomes, 20-40% of patients develop relapsed or refractory (R/R) disease with a dismal 2-year overall survival (OS) rate of only 20-40% (3). As a highly heterogeneous disease, the influence of patient metabolic status on the prognosis of DLBCL requires further elucidation.

Metabolic Syndrome (MetS) is a metabolic disorder characterized by the presence of at least three out of five components: obesity, hypertension, hyperglycemia, high levels of triglyceride (TG), and low levels of high-density lipoprotein cholesterol (HDL-C) (4). MetS not only increases the risk of cardiovascular and cerebrovascular diseases but also elevates the risk of cancer (5). Epidemiological studies have implicated components of MetS in the development and prognosis of hematologic malignancies. Nagel et al. reported that elevated glucose levels were associated with an increased risk of high-grade B-cell lymphomas and multiple myeloma, whereas reduced cholesterol levels were linked to low-grade B-cell lymphomas. In addition, a high BMI has been associated with an increased risk of Hodgkin lymphoma (6). Specifically, low levels of HDL-C have been shown to increase the risk of NHL and multiple myeloma (7, 8), while obesity and diabetes independently contribute to a higher incidence of DLBCL (9, 10). Beyond etiology, abnormalities in MetS components can also impact the effectiveness and prognosis of NHL. Studies have found a correlation between higher BMI and increased mortality risk in NHL patients (11, 12), and hyperglycemia and secondary hyperglycemia portended poor prognosis in DLBCL patients (13, 14). The impact of baseline lipid levels, including total cholesterol (TC), TG, HDL-C, and low-density lipoprotein cholesterol (LDL-C), before treatment and their subsequent changes on the prognosis of DLBCL remains unclear (15–17). Currently, no studies have shown a direct relationship between blood pressure and the effectiveness and prognosis of DLBCL.

Currently, there is a lack of longitudinal cohort studies systematically analyzing the relationship between dynamic changes in MetS components during treatment and the effectiveness and prognosis of DLBCL. Therefore, our study retrospectively established a longitudinal cohort of DLBCL patients who completed chemotherapy at our center. We analyzed the impact of baseline levels and dynamic changes in MetS components (obesity, blood pressure, blood glucose, lipids) on the treatment response and prognosis of DLBCL patients. This study aims to identify key metabolic indicators that may influence the effectiveness and prognosis of DLBCL, facilitating the integration of monitoring and managing MetS components into DLBCL treatment to improve patient outcomes.

## 2 Materials and methods

### 2.1 Patients

This retrospective cohort study included newly diagnosed DLBCL patients treated at the Department of Hematology, the First Affiliated Hospital of Dalian Medical University, between May 2010 to May 2022. Patients were eligible if they met the following criteria: (1) Age ≥ 18 years; (2) No history of liver disease or other malignant tumors; (3) Completed of 6–8 cycles of CHOP or CHOP-like regimen with or without rituximab at our center; (4) Availability of complete clinical and laboratory data. Exclusion criteria were: (1) Incomplete treatment cycles; (2) Missing treatment response evaluation; (3) Loss to follow-up.

Initially, 162 patients were enrolled, then 37 were excluded, resulting in a final cohort of 125 patients.

### 2.2 Data collection

Baseline demographic, clinical, and metabolic indicators were extracted from electronic medical records prior to the first treatment cycle. Variables included:

Demographics: Age, sex, height, weight, body mass index (BMI), systolic/diastolic blood pressure (SBP/DBP).
Laboratory indicators: Fasting plasma glucose (FPG), TG, TC, HDL-C, LDL-C.
DLBCL characteristics: Ann Arbor staging (I–IV), presence of B symptoms (fever, night sweats, and weight loss >10% within 6 months), International Prognostic Index (IPI) score, extranodal involvement, number of involved lymph node regions or extranodal sites, Hans classification (germinal center B-cell [GCB] vs. non-GCB subtype), and serum lactate dehydrogenase (LDH) levels.

During chemotherapy, clinical indicators, including hematologic and metabolic profiles, were collected before each chemotherapy cycle.

Treatment response was categorized as Complete Remission (CR), Partial Remission (PR), Stable Disease (SD), or Progressive Disease (PD) (18). For analytical purposes, outcomes were dichotomized into CR and Non-CR (PR/SD/PD) in this study.

Follow-up data was obtained through hospital records, outpatient visits, or telephone interviews until March 31, 2023. Progression-free survival (PFS) was defined as the interval from diagnosis to first disease progression, death, or last follow-up. Overall survival (OS) was defined as the interval from diagnosis to death or last follow-up.

### 2.3 Statistical analysis

All statistical analyses were performed using R software (version 4.3.2) and SAS 9.4. Continuous variables with normal distributions were presented as mean ± standard deviation (SD), whereas non-normally distributed variables were summarized as median with interquartile range (IQR). Categorical variables were described as frequency (percentage). Group comparisons were conducted using the independent samples t-test, Mann-Whitney U test, chi-square test or Fisher’s exact test as appropriate.

A multivariate logistic regression model was constructed with CR as the dependent variable, incorporating baseline demographic characteristics (age, sex), disease characteristics (Ann Arbor stage, GCB subtype, B symptoms, extranodal involvement, the number of involved areas, LDH levels and IPI score), and baseline metabolic components (FPG, TG, HDL-C, LDL-C, SBP, DBP, and BMI) as independent variables. A backward stepwise likelihood ratio test was employed to identify significant factors of treatment response. For survival outcomes, Cox proportional hazards regression was used to assess factors influencing progression-free survival (PFS) and overall survival (OS). The same set of independent variables was screened using backward stepwise likelihood ratio test for prognostic determinants.

Group-based trajectory modeling (GBTM) was implemented using the proc traj procedure in SAS 9.4 to characterize longitudinal trajectories of each MetS component during treatment (19). TG, HDL-C, and LDL-C underwent logarithmic transformation (*ln[value × 100*]) to normalize distributions. Optimal trajectory groups were selected based on the lowest Bayesian Information Criterion (BIC), an average posterior probability (AvePP) > 0.7, and subgroup proportion ≥ 5%.

Multivariate logistic regression analysis was performed with CR as the dependent variable. Independent variables included baseline demographic characteristics (age, sex), disease characteristics mentioned above, and trajectory groups of MetS components. A backward stepwise likelihood ratio test was applied to identify significant predictors of treatment response. For survival outcomes, Cox proportional hazards regression models were utilized to evaluate factors influencing PFS and OS. The same set of independent variables was analyzed using the backward stepwise likelihood ratio test to screen for prognostic determinants.

The proportional hazards assumption for Cox models was validated using the Schoenfeld residuals method and confirmed through global tests (*p* > 0.1 for all covariates). Collinearity between independent variables was assessed via variance inflation factors (VIFs), and the baseline TC was excluded from multivariate models due to its significant collinearity (VIF > 5). All statistical tests were two-tailed, with a significance level set at *p* < 0.05.

## 3 Results

### 3.1 Patient characteristics

This study followed 125 newly diagnosed DLBCL patients. Their median age at baseline was 61 years (IQR: 51 to 69 years), with 62 males (49.6%) and 63 females (50.4%). Nearly half of the patients (n=58, 46.4%) were diagnosed at an advanced stage (Ann Arbor III/IV). GCB subtype was identified in 38.4% (n=48) of patients, while 28.0% (n=35) exhibited B symptoms. Extranodal involvement was observed in 76.8% (n=96) of patients, with 42.4% (n=53) showing involvement of ≥3 lymph node regions or organs. Elevated LDH levels (>250 IU/L) were detected in 32.0% (n=40) of patients, and 32.8% (n=41) were categorized as high-intermediate risk or high risk (IPI ≥ 3). A total of 82 patients (65.6%) received the R-CHOP regimen, while 43 patients (34.4%) were treated with CHOP or CHOP-like regimens. CR was achieved in 71.2% (n=89) of patients following therapy. The data mentioned above, along with median values of the MetS components at baseline are presented in **Table 1**.

**Table 1:** Characteristics of patients with DLBCL at baseline.

Characteristics	N=125
Age, years	61 (51, 69)
Sex, female, n (%)	63 (50.4)
Ann Arbor stage III/IV, n (%)	58 (46.4)
GCB, n (%)	48 (38.4)
B symptoms, n (%)	35 (28.0)
Extranodal involvement, n (%)	96 (76.8)
Number of involved area ≥ 3, n (%)	53 (42.4)
LDH>250 IU/L, n (%)	40 (32.0)
IPI ≥ 3, n (%)	41 (32.8)
FPG, mmol/L	5.01 (4.69, 5.69)
TC, mmol/L	4.62 (3.76, 5.62)
TG, mmol/L	1.38 (0.94, 1.84)
HDL-C, mmol/L	1.07 (0.84, 1.31)
LDL-C, mmol/L	2.63 (2.06, 3.37)
SBP, mmHg	125.0 (117.0, 140.0)
DBP, mmHg	80.0 (70.0, 87.0)
BMI, kg/m^2^	24.34 (22.02, 26.54)

GCB, Germinal Center B-cell; LDH, Lactate Dehydrogenase; IPI, International Prognostic Index; FPG, Fasting Plasma Glucose; TC, Total Cholesterol; TG, Triglycerides; HDL-C, High-Density Lipoprotein Cholesterol; LDL-C, Low-Density Lipoprotein Cholesterol; SBP, Systolic Blood Pressure; DBP, Diastolic Blood Pressure; BMI, Body Mass Index.

Continuous variables were summarized as median with interquartile range (IQR).

### 3.2 Association between MetS components at baseline and DLBCL treatment response

As summarized in **Table 2**, statistically significant differences were observed between the CR and non-CR groups in Ann Arbor stage (*p* = 0.004), the number of involved area (*p*< 0.001), elevated LDH (*p* < 0.001), and IPI (*p* < 0.001). Baseline HDL-C level in the CR group was significantly higher than that in the non-CR group (*p* = 0.019). No significant differences were detected in other baseline MetS components between the two groups (*p* > 0.05). Univariate logistic regression analysis further confirmed that baseline MetS components were not associated with treatment response (*p* > 0.05), as presented in **Supplementary Table S1** (**Supplementary**).

**Table 2:** Comparison of baseline variables between CR and non-CR groups.

Baseline Variables	CR (n=89)	Non-CR (n=36)	*p*
Age	60 (51.0, 68.5)	63.5 (50.2, 70.5)	0.727
Sex, female, n (%)	48 (53.9)	15 (41.7)	0.214
Ann Arbor stage III/IV, n (%)	34 (38.2)	24 (66.7)	0.004
GCB, n (%)	37 (41.6)	11 (30.6)	0.251
B symptoms, n (%)	21 (23.6)	14 (38.9)	0.085
Extranodal involvement, n (%)	69 (77.5)	27 (75.0)	0.762
Number of involved area ≥ 3, n (%)	27 (30.3)	26 (72.2)	<0.001
LDH >250IU/L, n (%)	20 (22.5)	20 (55.6)	<0.001
IPI ≥3, n (%)	21 (23.6)	20 (55.6)	<0.001
FPG, mmol/L	5.01 (4.72, 5.64)	4.97 (4.59, 5.99)	0.764
TC, mmol/L	4.61 (3.76, 5.49)	4.67(3.81, 5.74)	0.482
TG, mmol/L	1.39 (0.85, 1.79)	1.35 (1.01, 2.05)	0.480
HDL-C, mmol/L	1.13 (0.90, 1.32)	0.90 (0.67, 1.27)	0.019
LDL-C, mmol/L	2.56 (2.06, 3.32)	2.72 (2.05, 3.44)	0.429
SBP, mmHg	126.0 (118.0, 140.0)	124.0 (111.8, 130.0)	0.158
DBP, mmHg	80(70.5, 87.5)	78.0 (70.0, 84.8)	0.261
BMI, kg/m^2^	24.22 (22.02, 26.54)	24.59 (21.98, 26.99)	0.645

GCB, Germinal Center B-cell; LDH, Lactate Dehydrogenase; IPI, International Prognostic Index; FPG, Fasting Plasma Glucose; TC, Total Cholesterol; TG, Triglycerides; HDL-C, High-Density Lipoprotein Cholesterol; LDL-C, Low-Density Lipoprotein Cholesterol; SBP, Systolic Blood Pressure; DBP, Diastolic Blood Pressure; BMI, Body Mass Index; CR, Complete Remission.

Continuous variables were summarized as median with interquartile range (IQR).

Backward stepwise multivariate logistic regression identified three independent influencing factors of CR: the number of involved areas (OR = 0.19, 95% CI: 0.09–0.50), LDH levels (OR = 0.38, 95% CI: 0.15–0.96, and baseline LDL-C (OR = 0.65, 95% CI: 0.44–0.97). Specifically, each one unit increase in baseline LDL-C was associated with a 35% reduction in the likelihood of achieving CR. Detailed results are provided in **Table 3**.

**Table 3:** Multivariate logistic regression for the association between baseline characteristics and treatment response.

Baseline Characteristics	Coef.	*p*	OR	OR 95%CI
Number of involved area ≥ 3	-1.660	0.001	0.19	(0.07, 0.50)
LDH > 250 IU/L	-0.959	0.044	0.38	(0.15, 0.96)
LDL-C, mmol/L	-0.430	0.033	0.65	(0.44, 0.97)

LDH, Lactate Dehydrogenase; LDL-C, Low-Density Lipoprotein Cholesterol; OR, Odds Ratio; CI, Confidence Interval.

### 3.3 Associations between MetS components at baseline and prognosis of DLBCL

The median follow-up time was 24 months (rang: 4–151 months, IQR:19–48 months), during which 14 patients (11.2%) experienced disease progression and 19 patients (15.2%) died. The estimated 2-year PFS and OS rate were 70.0% and 82.0%, respectively.

Univariate Cox regression analysis indicated that Ann Arbor stage, the number of involved area, LDH levels, IPI, and HDL-C were significant predictors of both PFS and OS (*p* < 0.05). An increase of one unit in baseline HDL-C was associated with a 64% decrease in the risk of progression (HR = 0.36, 95% CI: 0.15, 0.88) and an 82% reduction in the risk of death (HR = 0.18, 95% CI: 0.05, 0.65). Detailed hazard ratios are summarized in **Supplementary Tables S2** and **S3** (**Supplementary**).

The backward stepwise Cox regression model for PFS (**Table 4**) screened out three independent prognostic factors: the number of involved area ≥3 (HR = 3.85, 95% CI: 1.72–8.61), baseline HDL-C (HR = 0.27, 95% CI: 0.10–0.78) and baseline LDL-C (HR = 1.78, 95% CI: 1.14–2.79). An increase of one unit in baseline HDL-C was associated with an 73% reduction in progression risk. Conversely, an increase of one unit in baseline LDL-C raised the progression risk by 1.78 times. For OS, only the number of involved area ≥3(HR = 4.34, 95% CI: 1.34–14.10) and elevated LDH (HR = 2.77, 95% CI: 1.01–7.62) retained significance, with no independent associations observed for baseline MetS components.

**Table 4:** Multivariate cox regression for the impact of baseline characteristics on PFS.

Baseline Characteristics	Coef.	*p*	HR	HR 95%CI
Number of involved area ≥ 3	1.347	0.001	3.85	(1.72, 8.61)
HDL-C, mmol/L	-1.304	0.015	0.27	(0.10, 0.78)
LDL-C, mmol/L	0.577	0.011	1.78	(1.14, 2.79)

HDL-C, High-Density Lipoprotein Cholesterol; LDL-C, Low-Density Lipoprotein Cholesterol; HR, Hazard Ratio; CI, Confidence Interval.

### 3.4 Associations between MetS component trajectories and DLBCL treatment response

**Figure 1:**
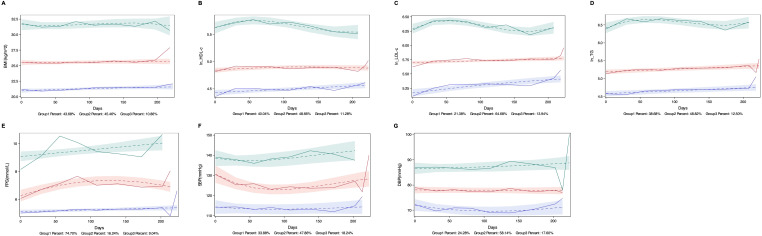
Trajectories of MetS Components. Low, medium, and high trajectory groups for each MetS Components are shown across treatment periods. **(A)** Body mass index (BMI): Low (20–22 kg/m²), medium (25–26 kg/m²; overweight range), and high (31–32 kg/m²; obesity range); **(B)** High-density lipoprotein cholesterol (HDL-C) (log-transformed as ln [HDL-C × 100]): Low (0.8–0.9 mmol/L), medium (1.2–1.3 mmol/L), and high (2.4–3.1 mmol/L); **(C)** Low-density lipoprotein cholesterol (LDL-C) (ln [LDL-C × 100]): Low (1.7–2.2 mmol/L), medium (2.9–3.0 mmol/L), and high (5.3–6.0 mmol/L); **(D)** Triglycerides (TG) (ln [TG × 100]): Low (0.9–1.0 mmol/L), medium (1.6–2.0 mmol/L), and high (6.0–7.3 mmol/L); **(E)** Fasting plasma glucose (FPG): Low (5.0–5.4 mmol/L), medium (6.2–7.3 mmol/L), and high (9.0–10.0 mmol/L); **(F)** Systolic blood pressure (SBP): Low (113–114 mmHg), medium (124–131 mmHg), and high (137–142 mmHg); **(G)** Diastolic blood pressure (DBP): Low (70–72 mmHg), medium (77–78 mmHg), and high (86–88 mmHg).

GBTM categorized longitudinal changes in each MetS component during treatment into three distinct trajectories: low-level, medium-level, and high-level groups. As illustrated in **Figure 1**, these trajectories remained stable relative to baseline values across all treatment cycles.

Univariate logistic regression (**Supplementary Table S4**) revealed that patients in the medium HDL-C trajectory group had a 3.32-fold higher likelihood of achieving CR compared to the low HDL-C group (OR= 3.32, 95% CI: 1.40–7.85). Similarly, the medium BMI trajectory group exhibited a 1.79-fold increased CR rate versus the low BMI group (OR = 2.79, 95% CI: 1.16–6.74).

Backward stepwise multivariate logistic regression analysis (**Table 5**) revealed that in addition to the number of involved area (OR = 0.26, 95% CI: 0.09–0.70) and LDH levels (OR = 0.33, 95% CI: 0.12–0.90) being significant predictors of treatment response, the LDL-C trajectory (*p* = 0.045) and BMI trajectory (*p* = 0.016) also significantly influenced treatment response. Compared to the low-level group, the high-level LDL-C trajectory group experienced a significant decrease in CR rate by approximately 90% (OR = 0.10, 95% CI: 0.01, 0.73), while the medium-level BMI trajectory group showed a 1.81-fold increase in CR rate (OR = 2.81, 95% CI: 1.00, 7.88).

**Table 5:** Multivariate logistic regression for the association between MetS components trajectory and treatment response.

Variables	Coef.	*p*	OR	OR 95%CI
LDH >250 IU/L	-1.111	0.031	0.33	(0.12, 0.90)
Number of involved area ≥3	-1.368	0.008	0.26	(0.09, 0.70)
LDLg		0.045		
Medium	0.095	0.856	1.10	(0.39, 3.08)
High	-2.345	0.023	0.10	(0.01, 0.73)
BMIg		0.016		
Medium	1.033	0.049	2.81	(1.00, 7.88)
High	-1.088	0.151	0.34	(0.08, 1.49)

LDH, Lactate Dehydrogenase; LDLg, Low-Density Lipoprotein Cholesterol trajectory;

BMIg, Body Mass Index trajectory; OR, Odds Ratio; CI, Confidence Interval.

### 3.5 Associations between MetS component trajectories and DLBCL prognosis

**Figure 2:**
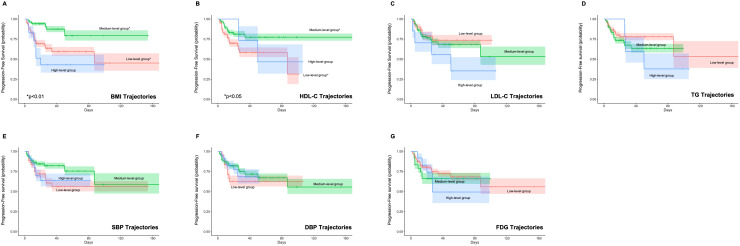
Impact of Metabolic Syndrome Component Trajectories on Progression-Free Survival (PFS) in DLBCL Patients. The association of **(A)** BMI (Body Mass Index), **(B)** HDL-C (High-Density Lipoprotein Cholesterol), **(C)** LDL-C (Low-Density Lipoprotein Cholesterol), **(D)** TG (Triglycerides), **(E)** SBP (Systolic Blood Pressure), **(F)** DBP (Diastolic Blood Pressure), and **(G)** FPG (Fasting Plasma Glucose) trajectories with PFS. BMI and HDL-C trajectories are associated with PFS, with medium-level trajectories showing improved outcomes.

**Figure 3:**
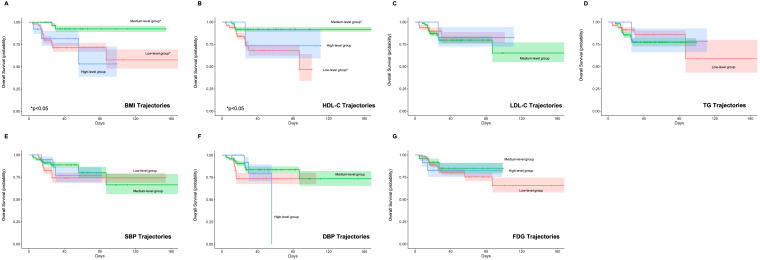
Impact of Metabolic Syndrome Component Trajectories on Overall Survival (OS) in DLBCL Patients. The association of **(A)** BMI (Body Mass Index), **(B)** HDL-C (High-Density Lipoprotein Cholesterol), **(C)** LDL-C (Low-Density Lipoprotein Cholesterol), **(D)** TG (Triglycerides), **(E)** SBP (Systolic Blood Pressure), **(F)** DBP (Diastolic Blood Pressure), and **(G)** FPG (Fasting Plasma Glucose) trajectories with OS. The medium BMI and HDL-C trajectory group shows a significant reduction in mortality risk compared to the low group.

Univariate Cox regression analysis revealed that longitudinal trajectories of HDL-C and BMI significantly influenced both PFS (**Figure 2**) and OS (**Figure 3**). Compared to the low HDL-C trajectory group, patients in the medium HDL-C trajectory group exhibited a 55% lower risk of disease progression (HR= 0.45, 95% CI: 0.21–0.93) and a 75% reduction in mortality risk (HR = 0.25, 95% CI: 0.08–0.76). Similarly, the medium BMI trajectory group showed a 71% decreased risk of disease progression (HR = 0.29, 95% CI: 0.12–0.69) and a 79% lower mortality risk (HR = 0.21, 95% CI: 0.06–0.75) compared to the low BMI trajectory group (**Supplementary Tables S5, S6**).

The backward Cox regression analysis identified BMI trajectory as an independent prognostic factor for both PFS and OS. For PFS, aside from the number of involved area (*p* < 0.001), the BMI trajectory (*p* = 0.012) was a significant predictor. Patients in the medium BMI trajectory group experienced a 67% reduction in progression risk compared to the low BMI trajectory group (HR = 0.33, 95% CI: 0.14, 0.78) (**Table 6**). In the OS analysis, along with the IPI (*p* = 0.030) and the number of involved area (*p* = 0.023), the BMI trajectory (*p* = 0.039) emerged as a crucial factor. The medium BMI trajectory group demonstrated an 81% reduction in mortality risk compared to the low BMI trajectory group (HR = 0.19, 95% CI: 0.05, 0.69) (**Table 7**).

**Table 6:** Multivariate cox regression for the relationship between MetS components trajectory and PFS.

Variables	Coef.	p	HR	HR 95%CI
Number of involved area ≥3	1.463	<0.001	4.32	(1.99, 9.38)
BMIg		0.012		
Medium	-1.112	0.012	0.33	(0.14, 0.78)
High	0.421	0.367	1.52	(0.61, 3.81)

BMIg, Body Mass Index trajectory; HR, Hazard Ratio; CI, Confidence Interval.

**Table 7:** Multivariate cox regression for the relationship between MetS components trajectory and OS.

Variables	Coef.	*p*	*HR*	*HR 95%CI*
IPI ≥ 3	-1.142	0.030	0.32	(0.11, 0.90)
Number of involved area ≥ 3	-1.371	0.023	0.25	(0.08, 0.83)
BMIg		0.039		
Medium	-1.653	0.011	0.19	(0.05, 0.69)
High	-0.193	0.767	0.83	(0.23, 2.95)

IPI, International Prognostic Index; BMIg, Body Mass Index trajectory; HR, Hazard Ratio;

CI, Confidence Interval.

## 4 Discussion

Our study demonstrated that BMI trajectories during treatment, rather than baseline BMI, served as critical predictors of both treatment response and survival. Patients with medium BMI levels during treatment had better prognosis compared to those with low BMI levels. Additionally, our findings highlight the dual role of lipid metabolism in DLBCL biology. While elevated baseline HDL-C is associated with favorable outcomes, elevated baseline LDL-C and persistently high LDL-C level independently predict reduced CR rate and increased progression risk. These results underscore the importance of monitoring and managing metabolic health throughout DLBCL therapy.

Previous studies have primarily focused on the relationship between BMI at diagnosis or baseline before treatment and the prognosis of DLBCL patients, without paying attention to the dynamic changes in BMI during treatment. A large study involving 1,386 DLBCL patients found that underweight patients at baseline had poorer prognoses compared to overweight and obese patients (20). Weiss et al. also supported this observation, showing that patients with BMI ≥ 25 kg/m² had a 3-year PFS of 74.1%, while those with a BMI < 25 kg/m² had a 3-year PFS of 57.5%; patients with BMI ≥ 25 kg/m² had a 3-year OS of 80.9%, compared to 64.2% for those with a BMI < 25 kg/m² (21). Another retrospective study of DLBCL patients among U.S. veterans showed that at diagnosis, patients with a BMI between 25 kg/m² and 29.9 kg/m² and those with a BMI ≥ 30 kg/m² had lower mortality risks compared to individuals with a normal BMI (22). We found that baseline BMI prior to therapy was not a significant factor affecting the treatment response and prognosis of DLBCL from both univariate and multivariate analyses. However, the BMI trajectory during treatment emerged as an important predictor of both outcomes. Patients maintaining a moderate BMI trajectory (25-26kg/m²) during therapy exhibited significantly higher CR rates and survival benefits (67% lower progression risk, 81% lower mortality risk) compared to those with low BMI trajectories. Overweight status may confer protective effects through the following mechanisms: Higher BMI provides a reserve of fat and muscle, which can help during treatment. Additionally, drug metabolism can be influenced by body composition, and higher BMI might increase drug concentration and treatment effectiveness (23). Obesity-related chronic low-grade inflammation may enhance immune system activity, improving therapy effectiveness (24). Moreover, higher BMI correlates with better nutritional status, aiding in tolerating treatment side effects (25). Lastly, higher BMI may be linked to favorable immune responses and beneficial tumor microenvironment changes (24).

Notably, the lack of association between high BMI trajectories (31–32 kg/m²) and treatment response and prognosis in our cohort likely reflects insufficient statistical power (10.9% subgroup proportion). Further studies with larger sample sizes are needed to clarify whether the high-level BMI trajectory exerts impacts on the treatment response and prognosis in DLBCL patients.

The impact of blood lipids on the prognosis of DLBCL remains inconclusive. A retrospective analysis of 271 R-CHOP-treated DLBCL patients demonstrated that concurrent statin therapy significantly improved CR rates PFS rate compared to non-statin users. This suggests that interfering with cholesterol metabolism pathways may enhance the sensitivity of lymphoma cells to chemotherapy (26). Additionally, reducing intracellular cholesterol in lymphoma cells has been shown to induce apoptosis (27). A retrospective study of 259 newly diagnosed DLBCL patients found a correlation between hyperlipidemia, particularly hypertriglyceridemia, and certain gene subtypes, but no correlation with prognosis (28).

Our analyses revealed complex relationships between lipid metabolism and treatment response in DLBCL. While baseline HDL-C levels were significantly higher in the CR group compared to non-CR group, multivariate logistic regression demonstrated that baseline LDL-C, rather than HDL-C, independently predicted lower CR rates. Notably, longitudinal LDL-C trajectories during treatment further exacerbated this association, and patients with persistently high LDL-C levels exhibited a 90% reduced probability of achieving CR compared to the low trajectory group. We also found that baseline HDL-C and LDL-C levels are important factors affecting the PFS of DLBCL patients. Elevated baseline HDL-C levels were favorable for extending PFS, while baseline LDL-C levels were detrimental. Furthermore, univariate analysis revealed a 56% reduction in progression rate and a 75% reduction in mortality rate in the medium-level HDL-C trajectory group compared to the low-level group. However, these associations lost significance in multivariate models, suggesting their prognostic impact may be mediated through other clinical variables.

A prospective study of 70 newly diagnosed DLBCL patients receiving (R)-CHOP treatment found that high HDL-C levels and low TG levels were protective factors against anthracycline-induced subclinical cardiotoxicity. This cardioprotective effect may partially explain the observed correlation between higher HDL-C levels and improved OS (29). Similarly, a retrospective study involving 307 newly diagnosed DLBCL patients treated with rituximab-containing regimens found high HDL-C predicted longer PFS and OS, whereas elevated TG was linked to shorter PFS (16). Our study did not find a correlation between TG levels and DLBCL prognosis, potentially due to limited sample size or shorter follow-up. A retrospective study of 46 transformed DLBCL patients reported that low HDL-C level was an independent prognostic predictor of poor OS in multivariate analysis (30). Another retrospective study of 367 newly diagnosed DLBCL patients treated with rituximab-containing regimens found that high baseline HDL-C and LDL-C were associated with favorable PFS and OS, also HDL-C or LDL-C elevations after 6–8 circles of chemotherapies were correlated with better survival (15), differing from our findings. Our application of GBTM to characterize longitudinal lipid dynamics during chemotherapy offers significant methodological advancements over prior approaches. Our trajectory analysis revealed that lipids exhibited remarkable stability during chemotherapy, with three distinct trajectory groups (“low,” “medium,” and “high”) maintaining near-baseline levels throughout treatment. This contrasts with prior studies that focused solely on binary pre-post treatment comparisons, which may overlook nuanced longitudinal patterns. Furthermore, GBTM accounts for intra-individual variability, reducing misclassification biases inherent in static measurements. These advantages underscore the importance of dynamic metabolic monitoring in optimizing DLBCL management.

Cholesterol and its derivatives contribute to a favorable tumor microenvironment by promoting inflammation and cellular proliferation, which are conducive to tumor growth (31, 32). HDL-C is known for its anti-inflammatory and antioxidant properties, which can help protect against cancer progression (33). HDL-C also facilitates cholesterol efflux from cells, which is crucial in maintaining cellular homeostasis and inhibiting tumor growth (34). HDL-C may enhance immune function, improve the body’s response to therapy, and inhibit the proliferation of cancer cells by modulating key signaling pathways (35). Elevated levels of LDL-C provide cancer cells with essential components for survival and growth, supporting altered cancer cell metabolism and facilitating membrane synthesis, hormone production, and cell signaling (36). Additionally, LDL-C can activate signaling pathways such as PI3K/AKT, which enhance cell survival and resistance to apoptosis, thereby may contributing to DLBCL aggressiveness (37). Oxidized LDL (oxLDL) further exacerbates these effects by inducing oxidative stress and inflammation, which promote cancer progression and potentially impair the effectiveness of chemotherapy (38). These mechanisms may help explain the findings of our study.

While retrospective studies have implicated preexisting diabetes mellitus (DM) as a risk factor for poor survival in DLBCL (39), and type 2 diabetes (DMT2) as an independent predictor of reduced PFS and OS (13), our cohort revealed no significant association between fasting plasma glucose (FPG) levels and treatment outcomes. The impact of glucocorticoid-induced diabetes during DLBCL treatment on patient prognosis remains controversial (14, 40). Current evidence on hypertension (HTN) and DLBCL prognosis is sparse and conflicting. A recent retrospective study involving 232 DLBCL patients found that arterial hypertension (AH) was an age-independent significant predictor of all-cause mortality and cardiovascular mortality (41). Our study did not find a significant impact of blood pressure on the prognosis of DLBCL patients. Larger multicenter cohorts with serial monitoring are needed to clarify whether glycemic or blood pressure control during therapy improves DLBCL outcomes.

This study has several limitations. First, the retrospective, single-center design inherently limits the generalizability of the results and introduces potential selection and information biases. Although rigorous inclusion criteria were applied, the relatively small cohort size (n = 125) may have restricted statistical power for subgroup analyses. Second, the median age of the cohort (58 years, IQR: 51–69) reflects an older population, which may reduce the applicability of these findings to younger DLBCL patients. Third, the extended enrollment period (2010–2022) and variable follow-up duration (range: 4–151 months) could introduce temporal biases. Notably, the short follow-up duration may underestimate long-term survival disparities associated with metabolic trajectories. Furthermore, the analysis did not fully account for interactions or mediating effects among MetS components and other clinical factors. Future studies employing machine learning algorithms or structural equation modeling could elucidate these complex relationships. To address these limitations, multicenter prospective cohorts with standardized metabolic monitoring protocols, extended follow-up duration, and sex or age-stratified analyses are strongly recommended.

## Data Availability

The raw data supporting the conclusions of this article will be made available by the authors, without undue reservation.
