# Supplementary material for: Association of dynamic changes in metabolic syndrome components with clinical outcomes in diffuse large B-cell lymphoma
Source: Front Oncol. 2025 Jun 16;15:1524498. doi: 10.3389/fonc.2025.1524498 (PMC12206803; doi:10.3389/fonc.2025.1524498)
Supplement: Supplementary file 1 [file Table1.docx]

Supplementary Material

**Supplementary Table 1. Univariate Logistic Regression for the Association Between Baseline Characteristics and Treatment Response**

| **Baseline Characteristics** | **Coef.** | ***p*** | **OR** | **OR 95%CI** |
| --- | --- | --- | --- | --- |
| Age | -0.000 | 0.999 | 1.00 | (0.97, 1.03) |
| Sex, female | 0.494 | 0.216 | 1.64 | (0.75, 3.59) |
| Ann Arbor stage III/IV | -1.174 | 0.005 | 0.31 | (0.14, 0.70) |
| GCB | 0.481 | 0.253 | 1.62 | (0.71, 3.69) |
| B symptoms | -0.723 | 0.088 | 0.49 | (0.21, 1.11) |
| Extranodal Involvement | 0.140 | 0.762 | 1.15 | (0.47, 2.84) |
| Number of involved area ≥3 | -1.787 | <0.001 | 0.17 | (0.07, 0.40) |
| LDH > 250 IU/L | -1.462 | <0.001 | 0.23 | (0.10, 0.53) |
| IPI ≥3 | -1.398 | <0.001 | 0.25 | (0.11, 0.56) |
| FPG, mmol/L | 0.087 | 0.631 | 1.09 | (0.76, 1.56) |
| TC, mmol/L | -0.122 | 0.304 | 0.89 | (0.69, 1.13) |
| TG, mmol/L | -0.268 | 0.100 | 0.77 | (0.56, 1.05) |
| HDL-C, mmol/L | 0.304 | 0.483 | 1.36 | (0.58, 3.17) |
| LDL-C, mmol/L | -0.233 | 0.199 | 0.79 | (0.56, 1.13) |
| SBP, mmHg | 0.019 | 0.134 | 1.02 | (0.99, 1.04) |
| DBP, mmHg | 0.028 | 0.183 | 1.03 | (0.99, 1.07) |
| BMI, kg/m^2^ | -0.066 | 0.218 | 0.94 | (0.84, 1.04) |

Abbreviations: GCB: Germinal Center B-cell, LDH: Lactate Dehydrogenase, IPI: International Prognostic Index, FPG: Fasting Plasma Glucose, TC: Total Cholesterol, TG: Triglycerides, HDL-C: High-Density Lipoprotein Cholesterol, LDL-C: Low-Density Lipoprotein Cholesterol, SBP: Systolic Blood Pressure, DBP: Diastolic Blood Pressure, BMI: Body Mass Index, OR: Odds Ratio, CI: Confidence Interval.

**Supplementary Table 2. Univariate Cox Regression for the Impact of Baseline** **Characteristics on PFS**

| **Baseline Characteristics** | **Coef.** | ***p*** | **HR** | **HR 95%CI** |
| --- | --- | --- | --- | --- |
| Age | 0.025 | 0.081 | 1.03 | (0.10, 1.06) |
| Sex, female | -0.268 | 0.444 | 0.76 | (0.38, 1.52) |
| Ann Arbor stage III/IV | 1.168 | 0.002 | 3.22 | (1.53, 6.78) |
| GCB | -0.074 | 0.836 | 0.93 | (0.46, 1.88) |
| B symptoms | 0.308 | 0.405 | 1.36 | (0.66, 2.81) |
| Extranodal involvement | -0.307 | 0.418 | 0.74 | (0.35, 1.55) |
| Number of involved area ≥ 3 | 1.528 | <0.001 | 4.61 | (2.14, 9.94) |
| LDH > 250 IU/L | 0.919 | 0.009 | 2.51 | (1.26, 4.99) |
| IPI ≥ 3 | 0.988 | 0.005 | 2.69 | (1.35, 5.33) |
| FPG, mmol/L | 0.051 | 0.721 | 1.05 | (0.77, 1.39) |
| TC, mmol/L | -0.047 | 0.648 | 0.95 | (0.78, 1.17) |
| TG, mmol/L | 0.072 | 0.488 | 1.07 | (0.88, 1.31) |
| HDL-C, mmol/L | -1.021 | 0.025 | 0.36 | (0.15, 0.88) |
| LDL-C, mmol/L | 0.037 | 0.797 | 1.04 | (0.78, 1.38) |
| SBP, mmHg | -0.006 | 0.578 | 0.99 | (0.97, 1.02) |
| DBP, mmHg | 0.005 | 0.786 | 1.01 | (0.97, 1.04) |
| BMI, kg/m^2^ | 0.073 | 0.126 | 1.08 | (0.98, 1.18) |

Abbreviations: GCB: Germinal Center B-cell, LDH: Lactate Dehydrogenase, IPI: International Prognostic Index, FPG: Fasting Plasma Glucose, TC: Total Cholesterol, TG: Triglycerides, HDL-C: High-Density Lipoprotein Cholesterol, LDL-C: Low-Density Lipoprotein Cholesterol, SBP: Systolic Blood Pressure, DBP: Diastolic Blood Pressure, BMI: Body Mass Index, HR: Hazard Ratio, CI: Confidence Interval.

**Supplementary Table 3. Univariate Cox Regression for the Impact of Baseline Characteristics on OS**

| **Baseline Data** | **Coef.** | **P** | **HR** | **HR 95%CI** |
| --- | --- | --- | --- | --- |
| Age | 0.035 | 0.067 | 1.04 | (0.10, 1.08) |
| Sex, male | -0.413 | 0.375 | 0.66 | (0.27, 1.65) |
| Ann Arbor stage III/IV | 1.635 | 0.004 | 5.13 | (1.67, 15.57) |
| GCB | -0.465 | 0.348 | 0.63 | (0.24, 1.66) |
| B symptoms | 0.609 | 0.192 | 1.84 | (0.74, 4.58) |
| Extranodal involvement | -0.286 | 0.564 | 0.75 | (0.28, 1.99) |
| Number of involved area ≥ 3 | 1.829 | 0.001 | 6.23 | (2.06, 18.86) |
| LDH > 250 IU/L | 1.525 | 0.002 | 4.59 | (1.79, 11.8) |
| IPI ≥ 3 | 1.469 | 0.002 | 4.34 | (1.69, 11.14) |
| FPG, mmol/L | -0.036 | 0.867 | 0.97 | (0.64, 1.46) |
| TC, mmol/L | -0.181 | 0.245 | 0.83 | (0.61, 1.13) |
| TG, mmol/L | 0.020 | 0.894 | 1.02 | (0.76, 1.37) |
| HDL-C, mmol/L | -1.743 | 0.009 | 0.18 | (0.05, 0.65) |
| LDL-C, mmol/L | -0.185 | 0.395 | 0.83 | (0.54, 1.27) |
| SBP, mmHg | 0.007 | 0.637 | 1.01 | (0.98, 1.04) |
| DBP, mmHg | 0.000 | 0.998 | 1.00 | (0.96, 1.05) |
| BMI, kg/m^2^ | 0.086 | 0.170 | 1.09 | (0.96, 1.23) |

Abbreviations: GCB: Germinal Center B-cell, LDH: Lactate Dehydrogenase, IPI: International Prognostic Index, FPG: Fasting Plasma Glucose, TC: Total Cholesterol, TG: Triglycerides, HDL-C: High-Density Lipoprotein Cholesterol, LDL-C: Low-Density Lipoprotein Cholesterol, SBP: Systolic Blood Pressure, DBP: Diastolic Blood Pressure, BMI: Body Mass Index, HR: Hazard Ratio, CI: Confidence Interval.

**Supplementary Table 4. Univariate Logistic Regression for the Relationship Between MetS Components Trajectory and Treatment Response**

|  | **Variables** | **Coef.** | ***p*** | **OR** | **OR 95%CI** |
| --- | --- | --- | --- | --- | --- |
| FPGg |  |  | 0.706 |  |  |
|  | Medium | 0.354 | 0.498 | 1.43 | (0.51, 3.97) |
|  | High | 0.405 | 0.561 | 1.50 | (0.38, 5.89) |
| TGg |  |  | 0.229 |  |  |
|  | Medium | -0.251 | 0.546 | 0.78 | (0.34, 1.76) |
|  | High | -1.410 | 0.087 | 0.24 | (0.05, 1.23) |
| HDLg |  |  | 0.024 |  |  |
|  | Medium | 1.198 | 0.006 | 3.32 | (1.40, 7.85) |
|  | High | 0.302 | 0.690 | 1.35 | (0.31, 5.96) |
| LDLg |  |  | 0.114 |  |  |
|  | Medium | 0.238 | 0.590 | 1.27 | (0.53, 3.02) |
|  | High | -1.371 | 0.092 | 0.25 | (0.05, 1.25) |
| SBPg |  |  | 0.575 |  |  |
|  | Medium | 0.415 | 0.347 | 1.51 | (0.64, 3.60) |
|  | High | 0.480 | 0.405 | 1.62 | (0.52, 5.00) |
| DBPg |  |  | 0.218 |  |  |
|  | Medium | 0.846 | 0.081 | 2.33 | (0.90, 6.02) |
|  | High | 0.606 | 0.293 | 1.83 | (0.59, 5.68) |
| BMIg |  |  | 0.008 |  |  |
|  | Medium | 1.028 | 0.022 | 2.79 | (1.16, 6.74) |
|  | High | -0.918 | 0.159 | 0.40 | (0.11, 1.43) |

Abbreviations: FPGg: Fasting Plasma Glucose trajectory, TGg: Triglycerides trajectory, HDLg: High-Density Lipoprotein Cholesterol trajectory, LDLg: Low-Density Lipoprotein Cholesterol trajectory, SBPg: Systolic Blood Pressure trajectory, DBPg: Diastolic Blood Pressure trajectory, BMIg: Body Mass Index trajectory, OR: Odds Ratio, CI: Confidence Interval.

**Supplementary Table 5. Univariate Cox Regression for the Impact of MetS Components Trajectory on PFS**

|  | **Variables** | **Coef.** | ***p*** | **HR** | **HR 95%CI** |
| --- | --- | --- | --- | --- | --- |
| FPGg |  |  | 0.637 |  |  |
|  | Medium | 0.370 | 0.373 | 1.45 | (0.64，3.27) |
|  | High | 0.280 | 0.609 | 1.32 | (0.45，3.87) |
| TGg |  |  | 0.840 |  |  |
|  | Medium | 0.217 | 0.557 | 1.24 | (0.60，2.56) |
|  | High | 0.155 | 0.813 | 1.17 | (0.32，4.22) |
| HDLg |  |  | 0.067 |  |  |
|  | Medium | -0.810 | 0.032 | 0.45 | (0.21，0.93) |
|  | High | -0.957 | 0.201 | 0.20 | (0.09，1.67) |
| LDLg |  |  | 0.605 |  |  |
|  | Medium | 0.103 | 0.798 | 1.11 | (0.50，2.44) |
|  | High | 0.597 | 0.328 | 1.82 | (0.55，6.00) |
| SBPg |  |  | 0.276 |  |  |
|  | Medium | -0.582 | 0.139 | 0.56 | (0.26，1.21) |
|  | High | -0.002 | 0.997 | 1.00 | (0.40，2.49) |
| DBPg |  |  | 0.789 |  |  |
|  | Medium | -0.281 | 0.492 | 0.76 | (0.34，1.68) |
|  | High | -0.199 | 0.709 | 0.82 | (0.29，2.32) |
| BMIg |  |  | 0.007 |  |  |
|  | Medium | -1.230 | 0.005 | 0.29 | (0.12，0.69) |
|  | High | 0.362 | 0.438 | 1.44 | (0.58，3.58) |

Abbreviations: FPGg: Fasting Plasma Glucose trajectory, TGg: Triglycerides trajectory, HDLg: High-Density Lipoprotein Cholesterol trajectory, LDLg: Low-Density Lipoprotein Cholesterol trajectory, SBPg: Systolic Blood Pressure trajectory, DBPg: Diastolic Blood Pressure trajectory, BMIg: Body Mass Index trajectory, HR: Hazard Ratio, CI: Confidence Interval.

**Supplementary Table 6. Univariate Cox Regression for the Impact of MetS Components Trajectory on OS**

|  | **Variables** | **Coef.** | ***p*** | **HR** | **HR 95%CI** |
| --- | --- | --- | --- | --- | --- |
| FPGg |  |  | 0.827 |  |  |
|  | Medium | -0.323 | 0.612 | 0.72 | (0.21，2.52) |
|  | High | 0.207 | 0.785 | 1.23 | (0.28，5.46) |
| TGg |  |  | 0.774 |  |  |
|  | Medium | 0.091 | 0.848 | 1.10 | (0.43，2.78) |
|  | High | -0.683 | 0.531 | 0.51 | (0.06，4.28) |
| HDLg |  |  | 0.032 |  |  |
|  | Medium | -1.387 | 0.015 | 0.25 | (0.08，0.76) |
|  | High | -1.336 | 0.201 | 0.26 | (0.03，2.04) |
| LDLg |  |  | 0.809 |  |  |
|  | Medium | 0.010 | 0.985 | 1.01 | (0.38，2.70) |
|  | High | -0.670 | 0.541 | 0.51 | (0.06，4.39) |
| SBPg |  |  | 0.760 |  |  |
|  | Medium | -0.364 | 0.466 | 0.70 | (0.26，1.85) |
|  | High | -0.265 | 0.697 | 0.77 | (0.20，2.91) |
| DBPg |  |  | 0.550 |  |  |
|  | Medium | -0.553 | 0.284 | 0.58 | (0.21，1.58) |
|  | High | -0.487 | 0.494 | 0.61 | (0.15，2.48) |
| BMIg |  |  | 0.046 |  |  |
|  | Medium | -1.541 | 0.016 | 0.21 | (0.06，0.75) |
|  | High | 0.102 | 0.874 | 1.11 | (0.32，3.89) |

Abbreviations: FPGg: Fasting Plasma Glucose trajectory, TGg: Triglycerides trajectory, HDLg: High-Density Lipoprotein Cholesterol trajectory, LDLg: Low-Density Lipoprotein Cholesterol trajectory, SBPg: Systolic Blood Pressure trajectory, DBPg: Diastolic Blood Pressure trajectory, BMIg: Body Mass Index trajectory, HR: Hazard Ratio, CI: Confidence Interval.
